# Buschke-Löwenstein tumor of the vulva in a patient with a history of squamous cell carcinoma of the cervix

**DOI:** 10.1186/1471-2334-14-S7-P11

**Published:** 2014-10-15

**Authors:** Maria Isabela Sarbu, Ion Filimon Sarbu, Mircea Tampa, Vasile Benea, Ilinca Nicolae, Clara Matei, Teodor Poteca, Simona Roxana Georgescu

**Affiliations:** 1 Clinical Hospital of Infectious and Tropical Diseases “Dr. Victor Babeş”, Bucharest, Romania; 2 Schuller Municipal Hospital, Ploieşti, Romania; 3 Carol Davila University of Medicine and Pharmacy, Bucharest, Romania; 4 Colentina Clinical Hospital, Bucharest, Romania

## Background

Buschke-Löwenstein tumor (BLT) is a very rare sexually-transmitted disease associated with human papillomavirus (HPV) type 6 and 11, but rare cases of oncogenic HPV types including HPV 16 and HPV 18 were also reported. It is characterized by slow, locally invasive growth and clinically presents as exophytic masses with a cauliflower-like morphology. It usually occurs in uncircumcised men and location in sites other than the penis is very rare. It is a premalignant disorder with a low incidence of metastasis but with a considerable risk of malignant transformation to squamous cell carcinoma (SCC).

## Case report

We report the case of a 51-year-old female patient who addressed our clinic for a large, exophytic, cauliflower-like tumor involving the vulva and the perineum. The patient had been diagnosed with invasive SCC of the cervix 13 years before and had undergone intracavitary brachytherapy with Iridium-192 with a total dose of 60 Gy at point A, followed by total hysterectomy with bilateral salpingo-oophorectomy and lymphadenectomy. She showed no recurrences to this day. At the same time as the cervical SCC diagnosis the patient presented small, papular, erythematous lesions located on the vulva and had been diagnosed with condyloma acuminata. She underwent several conservative therapies including podophyllin, cryotherapy and electrocoagulation as well as debulking and surgical treatment with several relapses over the years. The patient had not used any treatment for three years before returning to our department, during which time the lesions had slowly grown reaching giant dimensions.

Several biopsies were taken from the tumor and confirmed the clinical diagnosis of Buschke-Löwenstein tumor. The HIV testing turned out negative. The patient tested positive for HPV genotype 16. The other laboratory tests were within normal range.

## Conclusion

Buschke-Löwenstein tumor can be associated with a high rate of recurrence and a risk of malignant transformation to invasive SCC, especially in patients with oncogenic types of HPV. The patient was sent to the general surgery department for excision and remains under the supervision of the dermatology and oncology department for rapid treatment of relapses and early detection of malignant transformation.

## Consent

Written informed consent was obtained from the patient for publication of this Case report and any accompanying images. A copy of the written consent is available for review by the Editor of this journal.

